# Unravelling the Mystery of Stem/Progenitor Cells in Human Breast Milk

**DOI:** 10.1371/journal.pone.0014421

**Published:** 2010-12-28

**Authors:** Yiping Fan, Yap Seng Chong, Mahesh A. Choolani, Mark D. Cregan, Jerry K. Y. Chan

**Affiliations:** 1 Experimental Fetal Medicine Group, Department of Obstetrics and Gynaecology, Yong Loo Lin School of Medicine, National University Health System, Singapore, Singapore; 2 School of Biomedical, Biomolecular and Chemical Sciences, The University of Western Australia, Crawley, Perth, Australia; 3 Department of Reproductive Medicine, KK Women's and Children's Hospital, Singapore, Singapore; 4 Cancer and Stem Cell Biology Program, Duke-NUS Graduate Medical School, Singapore, Singapore; Istituto Dermopatico dell'Immacolata, Italy

## Abstract

**Background:**

Mammary stem cells have been extensively studied as a system to delineate the pathogenesis and treatment of breast cancer. However, research on mammary stem cells requires tissue biopsies which limit the quantity of samples available. We have previously identified putative mammary stem cells in human breast milk, and here, we further characterised the cellular component of human breast milk.

**Methodology/Principal Findings:**

We identified markers associated with haemopoietic, mesenchymal and neuro-epithelial lineages in the cellular component of human breast milk. We found 2.6±0.8% (mean±SEM) and 0.7±0.2% of the whole cell population (WCP) were found to be CD133+ and CD34+ respectively, 27.8±9.1% of the WCP to be positive for Stro-1 through flow-cytometry. Expressions of neuro-ectodermal stem cell markers such as nestin and cytokeratin 5 were found through reverse-transcription polymerase chain reaction (RT-PCR), and in 4.17±0.2% and 0.9±0.2% of the WCP on flow-cytometry. We also established the presence of a side-population (SP) (1.8±0.4% of WCP) as well as CD133+ cells (1.7±0.5% of the WCP). Characterisation of the sorted SP and non-SP, CD133+ and CD133- cells carried out showed enrichment of CD326 (EPCAM) in the SP cells (50.6±8.6 vs 18.1±6.0, *P*-value  = 0.02). However, culture in a wide range of in vitro conditions revealed the atypical behaviour of stem/progenitor cells in human breast milk; in that if they are present, they do not respond to established culture protocols of stem/progenitor cells.

**Conclusions/Significance:**

The identification of primitive cell types within human breast milk may provide a non-invasive source of relevant mammary cells for a wide-range of applications; even the possibility of banking one's own stem cell for every breastfeeding woman.

## Introduction

The mammary gland is metabolically active, and has the capacity to undergo cycles of extensive proliferation and hypertrophy in order to meet the needs of pregnancy, lactation and involution. In order to support this activity, the presence of mammary stem cells (MaSC) which have the ability to give rise to different components of the lactation machinery have been proposed.

The existence of MaSC was first demonstrated nearly five decades ago with the successful reconstitution of the entire murine mammary gland through the transplantation of non-sorted mammary epithelium into cleared fat pads [1], although the identity of the components of the epithelium transplanted wasn't well defined. More recently, Gudjonsson et al, using well characterised immortalized cell lines from human breast tissues, demonstrated that these mammary stem cells are derived from the suprabasal compartment of the ductal epithelium in human breast [2]. This was followed closely by the description of a serum-free spheroid culture system which enriches for MaSC that demonstrated self-renewal and the capacity to differentiate into terminal ductal lobular units (TDLU) when placed into matrigel-coated plates, allowing their culture and *in vitro* behavior to be studied [3].

The prospective isolation of MaSC capable of reconstituting mammary glands was first demonstrated by Alvi et al, who identified these cells by their ability to exclude Hoechst dye [4]. Finally, Shackleton et al demonstrated the reconstitution of an entire mammary gland from a single lineage negative, CD29hi and CD49^+^ murine mammary cell, which were capable of generating secondary clonal outgrowths in serial transplantation experiments, conclusively demonstrating the existence of MaSC [5].

The derivation of both normal MaSC [3], [5] and breast cancer stem cells [6], [7] should allow the delineation of molecular pathways implicated in breast cancer oncogenesis and prognostication applications [8].

Despite the proximity of epidermal stem cell niches to their luminal cavities, there have been few studies documenting their presence in luminal discharges. In the gastro-intestinal system, stem cells have been localized to the basal crypts [9], [10], although there have been no reports of these epithelial stem cells being shed into the gastrointestinal tract. Similarly, it has been proposed that the epithelial stem cells reside in the niche at the base of the glands in the endometrium [11], and shown to be present just beneath the luminal epithelium and in the endometrial-myometrial junction [12], [13]. More recently, mesenchymal progenitor cell types have been isolated through the collection of human menstrual blood as well as human breast milk (HBM) [14], [15], [16]. In the bladder, rare stem/progenitor cell types from the epithelial, urothelial and smooth muscle lineage have been identified at a clonal level, with the capacity for self-renewal and multi-lineage differentiation [17].

Breast milk comprises epithelial cells, colostral corpuscles, polymorphonuclear leukocytes, mononuclear phagocytes and lymphocytes [18], [19], with those of epithelial lineage forming the main bulk of cells within two weeks of establishing lactation [20]. We hypothesised that these epithelial cells are shed from the ductal and luminal epithelial layers through either a heightened turnover of the secretory tissue, or as a consequence of the mechanical shear forces associated with the continued filling and emptying cycle associated with breast milk synthesis and lactation. We have previously identified putative MaSC from HBM through their expression of various cytokeratin (CK) markers, CK5, 14 and 19 and nestin [21], but have yet to establish other hallmarks of stem/progenitor cells. In this study, we isolated putative stem cell populations in HBM, and characterise their potential to self-renew and differentiate down various lineages, in order to establish their identity as stem/progenitor cells.

## Results

### Breast milk contains a heterogeneous population of cells derived from various lineages

**Figure 1:**
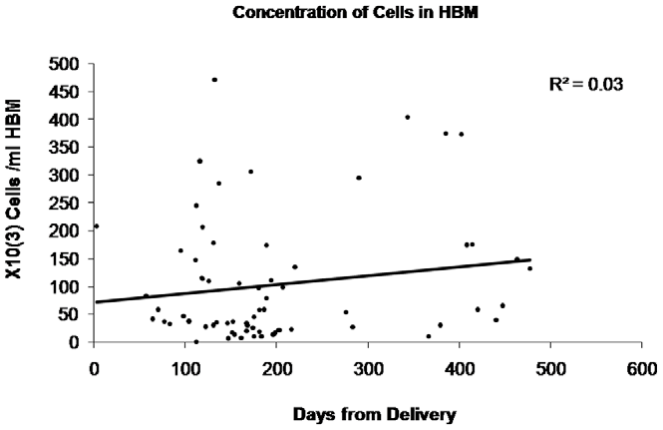
Cellular concentration in human breast milk did not vary in relation to the duration of breastfeeding.

The cell concentration in milk ranges widely from 1×10^3^ to 8×10^5^ cells per ml of milk, which was not related to the duration of lactation (r^2^ = 0.03, Fig. 1). The cellular components included a heterogenous population of cells comprising neutrophils, lymphocytes, monocytes, lactocytes and macrophages as was previously described [22]. In order to further characterise this heterogeneous cellular population of HBM, we looked for lineage specific markers in this mixed cell population at the mRNA and protein level in freshly isolated, uncultured WCP from HBM.

### Haemopoietic stem cell markers exists in uncultured WCP in HBM

**Figure 2:**
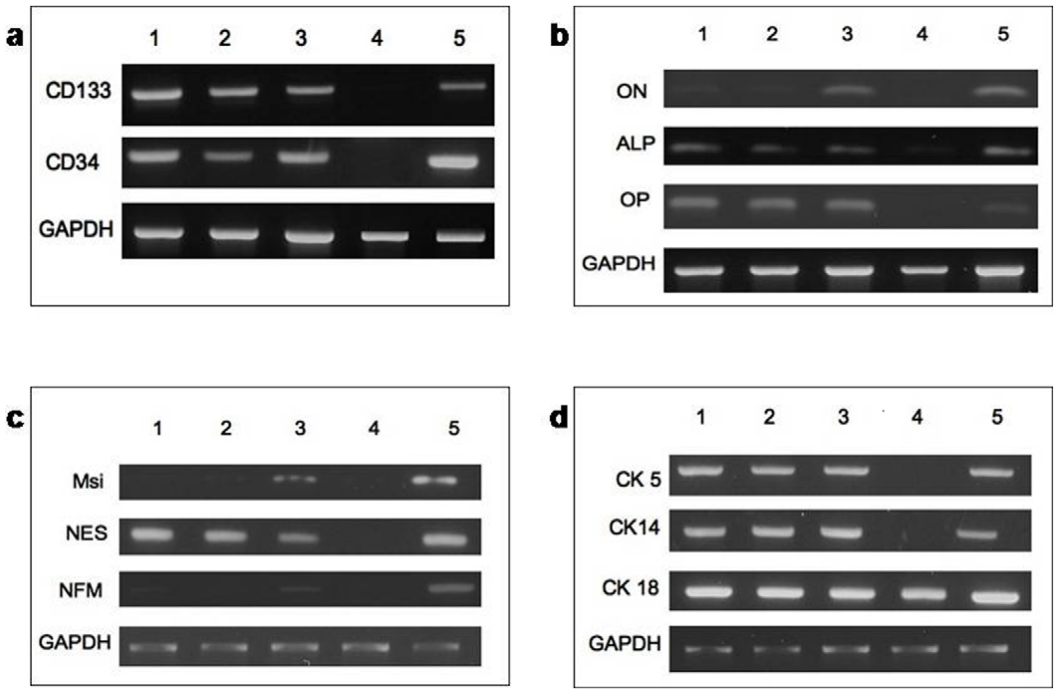
RT-PCR on Messenger RNA (mRNA) of milk samples from three individuals. (a) mRNA of CD133 and CD34 were present in WCP of HBM (Lane 1–3). (b) Osteonectin (ON), alkaline phosphatase (ALP) and osteopontin (OP) (Lane 1–3) as well as (c) musashi-1 (Msi), nestin (NES) and neurofilament-M (NFM) in observed in WCP of HBM (Lane 1–3). (d) Messenger RNA of CK5, 14 and 18 were present in WCP of HBM (Lane 1–3). The negative controls in Lane 4, were MCF-7 for hematopoietic, mesenchymal and neural markers [49] and mononuclear cells in peripheral blood for epithelial cell markers of CK5 and CK14. Positive controls (Lane 5) are cells isolated from umbilical cord blood (a), fetal MSC (b), cells from snap-frozen fetal brain (c) and MCF-7 (d) respectively.

First, we looked for the presence of haemopoietic stem/progenitor cell types through the presence of CD34, a well known haemopoietic stem cell marker [23], and CD133, which is associated with haemopoietic as well as neural stem/progenitor cells [24], [25]. Reverse transcription polymerase chain reaction (RT-PCR) demonstrated the presence of CD34 and CD133 mRNA transcripts in all three WCP samples examined, but not in a breast adenocarcinoma cell line, MCF-7 (Fig. 2a). Flow cytometry performed in a separate experiment (n = 4) showed a mean of 2.6 ± 0.79% and 1.1 ± 0.15% of the WCP being CD133 and CD34 positive respectively (Table 1).

**Table 1:** Antigens expressed on the cells in HBM.

Antigen	Name	Expression determined by Flow Cytometry		
		Mean (%)	No. of Samples	S.E.M.
**CD133**	Prominin-like 1	2.6	4	0.8
**CD117**	c-kit	0.0	3	0.0
**CD24**	BA-1	70.7	3	10.3
**CD29**	β-1 Integrin	11.5	3	5.8
**CD49f**	Α-6 Integrin	11.4	3	7.3
**CDw338**	ABCG2	21.1	3	1.8
**CD34**		0.7	3	0.3
**Stro-1**		27.8	3	9.1
**Nestin**		4.7	3	0.2

### Cells of mesenchymal lineages exist in uncultured WCP in HBM

Next, we looked for the presence of mesenchymal lineages in WCP. We found that transcripts for osteonectin, alkaline phosphatase and osteopontin, relating to osteogenic differentiation, were expressed in all samples tested (n = 3) (Fig. 2b) [26]. In addition, flow cytometry demonstrated the presence of Stro-1, a well known marker for mesenchymal stem cells (MSC) [27], in 27.8 ± 9.13% of the WCP (n = 3), suggesting that mesenchymal osteo-progenitors may reside within this mixed population.

### Neuro-epithelial lineage in uncultured WCP in HBM

Finally, we looked for the presence of transcripts associated with neuro-epithelial tissues in WCP. We found uniform expression of nestin, an early neural progenitor marker, in all samples examined, but only one out of three samples expressing both Musashi-1 (Msi-1) [28] and neurofilament M (NFM) [29] (Fig. 2c), markers of early and late neural differentiation respectively. This suggested the presence of both differentiated and undifferentiated neuro-progenitor cell types in this sample.

Cytokeratin (CK) 5, CK14 and CK18 are established markers used to delineate the degree of differentiation of mammary epithelial cells. CK5 is a marker for mammary progenitor cells, CK14 is a marker for both mammary progenitor cells and mature myoepithelial cells and CK18 is an established marker for mature luminal epithelial cells [30]. RT-PCR of WCP demonstrated universal expression of CK5, CK14 and CK18 in all samples examined (n = 3) (Fig. 2d).

### Side-population of HBM is enriched for primitive cell markers

**Figure 3:**
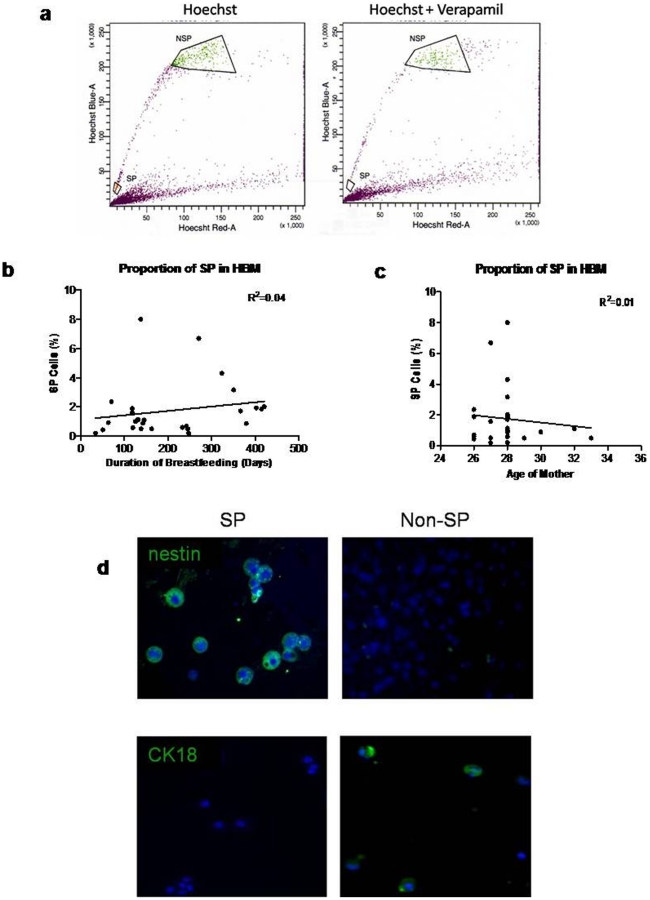
Side-population of HBM. (a) Sorting profile of side-population (SP) and non side-population (NSP) from breast milk. (b) Scatter plots showing no relationship between the percentage of SP and duration of breastfeeding and (c) age of mother. (d) Immunocytochemistry illustrating expression of nestin exclusively only on SP (top left) and expression of CK 18 exclusively on NSP (bottom right).

We next determine the proportion of cells that are able to exclude Hoechst 33342 dye in 47 mothers, and explored their capacity for self-renewal and differentiation. We found the existence of a SP in 40 out of 47 milk donors (85.1%) (Fig. 3a), at a frequency of 2.06±0.33%, which was not related to either the duration of breastfeeding or age of the mother (Fig. 3b, c). After flow-sorting SP and non-SP (NSP) cells, the sorted cells were stained for the expression of various epithelial and non-epithelial markers (Table 2).

**Table 2:** Antigens expressed on the SP and NSP in HBM.

Properties	Antigen	Expression on SP	Expression on NSP	P-value
	CD44	5.9±2.4	23.3±10.0	0.25
**Mammary**	CD24	47.1±19.1	37.5±17.7	0.42
**Stem Cell**	CD29	0.3±0.2	2.7±1.9	0.36
	CK5	9.4±4.4	12.0±6.0	0.79
**Mammary**	CD10	3.9±2.3	1.7±0.7	0.53
**Epithelial Cell**	EPCAM	50.6±8.6	18.1±6.0	0.02*
	CD45	5.7±2.3	12.7±4.6	0.25
	Lin	37.8±17.8	5.7±1.5	0.29
**Non-**	CD105	27.9±9.9	6.5±4.4	0.32
**Epithelial**	CD31	1.9±0.2	0.7±0.3	0.04*
	Stro-1	11.5±8.7	2.1±0.8	0.38
	CD73	0.1±0.04	0.0±0.04	0.30

**Figure 4:**
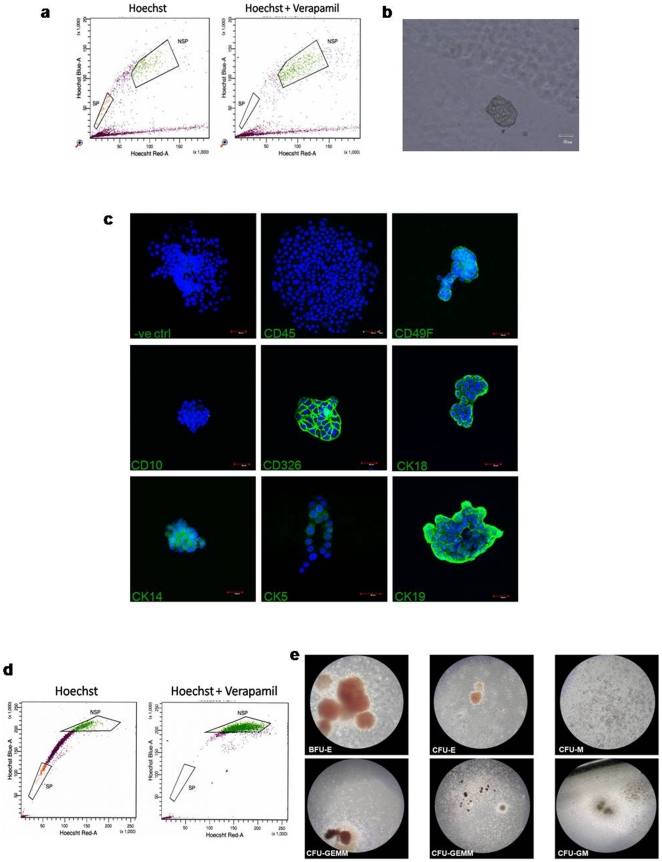
Culture of SP of positive control. (a) Flow cytometry profile of MCF-7, a breast cancer cell line from which the side population which was sorted out (b) grew into mammospheres after 10 days with (c) immunocytochemistry of the mammospheres ascertaining their identity. (d) Flow cytometry profile of umbilical cord blood with the side-population sorted out for culture in methylcellulose, (e) confirming the presence of burst forming unit-erythrocyte (BFU-E), colony forming unit-erythrocyte (CFU-E), colony forming unit-macrophage (CFU-M), colony forming unit-granulocyte, erythrocyte, macrophage, megakaryocyte (CFU-GEMM) as well as colony forming unit-granulocyte, macrophage (CFU-GM) in the SP of cord blood.

The mature marker CK18 was expressed by a larger proportion of cells in non-SP compared to SP cells (39.4 ± 9.8% NSP vs. 5.3 ± 4.0% NSP cells, p<0.05), while there was a higher occurrence of primitive nestin+ cells in the SP over the NSP population (90 ± 4.7% of SP vs 0.67 ± 0.54% of non-SP, p<0.001) (Fig. 4d). EPCAM, which is known to be expressed by bi-potent mammary progenitor cells, which give rise to EPCAM+ luminal progenitors and EPCAM- myoepithelial progenitors [31], [32], was found to be enriched in the SP fraction (50.6±8.6% vs 18.1±6.0%, p = 0.02). We found a small increase in CD31+ cells in the SP (1.9±.2% vs. 0.7±0.3%, p = 0.04) over the NSP fraction, which may indicate the presence of haemopoietic and/or endothelial cells, which are known to express CD31 [33], within the SP (Table 2).

### Side-population of HBM did not contain mammary stem cells or haemopoietic stem/progenitor cells or mesenchymal stem cells

In order to better define putative stem/progenitor cells, we attempted to culture SP fraction as mammospheres or serum-free spheroid culture system, which has been largely used to define the self-renewal capacity of MaSC in both normal and malignant mammary tissues [3], [34], [35], [36], [37], [38], [39]. Despite plating SP cells at a range of plating densities, addition of varying concentrations of growth factors (20–00 ng/ml of EGF and bFGF) and using the aid of different substratum, no mammospheres was observed (See supplementary data). On the other hand, serum-free culture of the SP (Fig. 4a) and not the NSP of a breast carcinoma cell line, MCF-7 resulted in the generation of free-floating mammospheres (Fig. 4b). These MCF-7 SP derived mammospheres stained positive for CD49f, EPCAM, CK18, CK14, CK5 and CK19, and negative for CD45 and CD10, an immunophenotype consistent with MaSC cultured as mammospheres (Fig. 4c).

As the SP fraction in bone marrow is enriched for haemopoietic stem cells [40], we next interrogated HBM for the presence of haemopoietic stem cell types by culturing SP and NSP fractions of HBM in a methylcellulose culture system. However, we did not find the expansion of any haemopoietic colonies (n = 5) in either SP or NSP fraction from HBM, while we observed the development of multi-lineage colony forming units (CFU) from the SP fraction of umbilical cord blood (Fig. 4e). Thus, both the SP and NSP fractions of HBM did not contain any haemopoietic progenitors that behave in the typical manner of haemopoietic progenitors.

**Figure 5:**
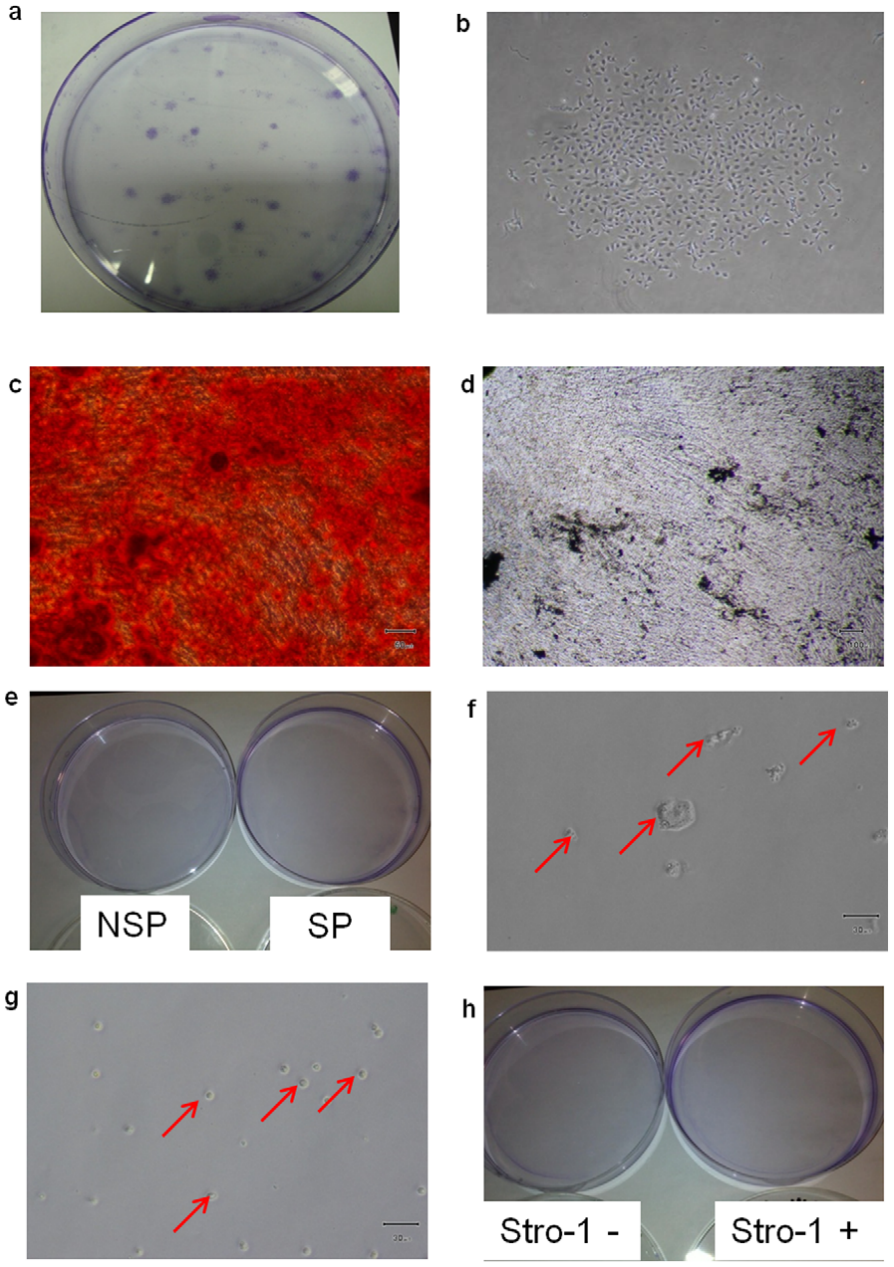
Mesenchymal Culture of Cells. (a, b) Adherent colonies of fetal MSC emerged after low density seeding at 4 cells per cm^2^. Osteogenic induction of fetal MSC resulted in the deposition of extracellular calcium crystals staining positive with Alizarin red (c) and Von Kossa (d).CFU-F assays of SP and NSP as well as Stro1 positive and negative fractions of HBM did not establish any colonies as shown by the absence of colonies (e, h). WCP (f) and Stro-1 positive cells (g) cultured in D10 remained as non-adherent cells (red arrows) which did not undergo any proliferation in culture.

Next, we investigated whether the SP or NSP fractions enriched for putative MSC in HB (n = 3 individual samples) [16]. Comparing the SP and NSP fraction for two MSC markers [41] and Stro-1, we found no differences between the two fractions in their expression of CD73, CD105 and Stro-1 (Table 2). We also investigated the ability of the two fractions to give rise to colony-forming unit-fibroblast (CFU-F), alongside the positive control of human fetal MSC. Only human fetal MSC were able to form adherent colonies in standard MSC CFU-F assay (Fig. 5a, b) and differentiated down the osteogenic pathway as evidenced by the accumulation of extracellular calcium crystals stained positively with Alizarin Red and Von Kossa (Fig. 5c, d), but not in either SP or NSP fractions from HBM of three individuals (Fig. 5e).

### CD133-positive cells of HBM did not contain mammary stem cells or haemopoietic stem/progenitor cells

**Figure 6:**
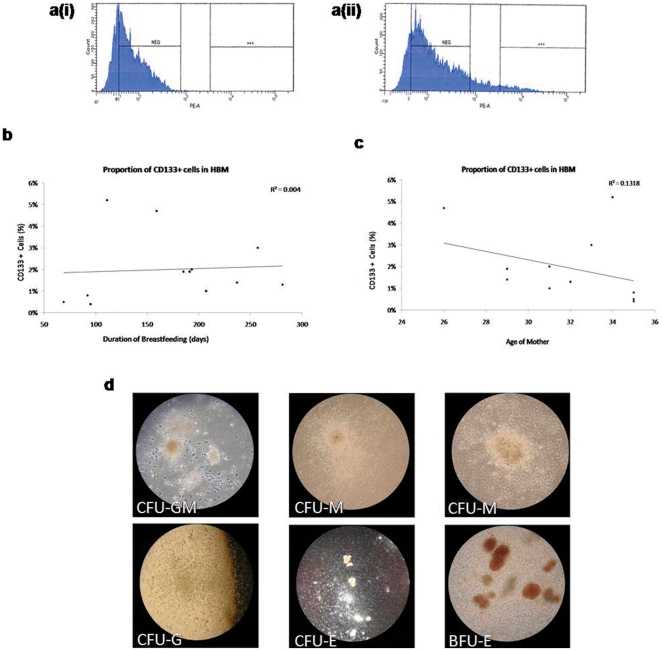
CD133 Sorting. (a) Flow cytometry of CD133 staining on cellular component of HBM (i), as compared to the isotype control (ii). (b, c) Three was no relationship between the frequency of CD133 cells and the duration of breastfeeding, nor the age of the mother. (d) CD133+ cells from cord blood formed multi-lineage colonies on CFC assays.

CD133 has been associated with several different primitive cell populations, such as haemopoietic, neural, endothelial stem/progenitor cells, and other primitive cells [42]. Since it is a primitive cell marker present on haemopoietic and neuro-ectodermal stem/progenitor cells [24], [25], we hypothesised that the putative stem cells in HBM may be found in the CD133 fraction of HBM. We found that 2.0±0.003% (n = 23) of the cellular component in HBM are CD133+ (Fig. 6a), which was unaffected by either the maternal age or the duration of breastfeeding (Fig. 6b, c).

CD133+ HBM cells did not demonstrate any significant differences in their expression of EPCAM, CD34, CD45, lineage and CD105 with CD133- HBM cells (Table 3). When plated onto methylcellulose cultures for haemopoietic assays, umbilical cord blood CD133+ cells, and not HBM CD133+ cells, demonstrated multi-lineage haemopoietic colony forming capacity (Fig. 6d). Both CD133+ and CD133− HBM cells did not generate any mammospheres in serum free growth conditions.

**Table 3:** Antigens expressed on CD133+ and CD133- cells in HBM.

Properties	Antigen	Expression on CD133+	Expression on CD133−	P-value
**Mammary**	EPCAM	1.9±1.0	1.3±0.5	0.63
	CD45	4.2±1.9	8.2±2.1	0.21
**Non-**	Lin	0.5±0.2	1.1±0.6	0.36
**Epithelial**	CD105	2.8±1.3	11.7±1.8	0.06
	CD31	0±0	0.48±0.4	0.36

### Mensenchymal culture of cells in HBM

In order to isolate putative MSC from HBM, we pursued two approaches. Firstly, WCP from HBM (n = 4) was plated in MSC growth medium (D10) at densities of 5×10^4^ to 14×10^4^ cells per cm^2^, with half medium change every four days. In contrast to human fetal MSC controls, which generated adherent colonies over 14 weeks of culture and differentiated into osteoblasts staining positive with Alizarin Red and Von Kossa, no adherent cultures were seen in the WCP (Fig. 5f).

Next we attempted to enrich the putative MSC cells from WCP through expression of the osteoprogenitor marker Stro-1 by flow cytometry (n = 3). We did not select Stro-1 cells from the SP fraction as there was no difference in the frequency of Stro-1 positive cells in either SP or NSP fraction (Table 2). Both Stro-1 positive and negative cells from WCP of HBM were seeded in D10 at a cell density ranging from 10×10^3^ to 36×10^3^ to cells per cm^2^. However, no fibroblastic adherent colonies were established (Fig. 5g). Colony-forming unit assays with either Stro-1 positive or negative cells showed no formation of colonies (Fig. 5h).

## Discussion

Here, we demonstrated that cells with markers associated with haemopoietic, mesenchymal as well as neuro-epithelial lineages are found in the cellular component of HBM. The findings of the numerous markers associated with haemopoietic, mesenchymal and neuro-epithelial markers at both mRNA and protein level confirmed the heterogeneity of uncultured HBM. The positive findings of CK5, CK14 and CK18 mRNA transcripts in all 3 of the samples tested contrasted with our previous data in which CK5 was not identified in WCP, but was expressed only after two weeks of in vitro culture in lactocyte medium till confluency [21]. As these three markers are well-established to delineate the varying stages of differentiation [30], we proposed that the disparity observed is likely to be due to the highly heterogenous nature of HBM, as illustrated earlier by the differential expression of neural markers, Msi-1 and NFM.The positive expression of various primitive stem/progenitor cell markers like CD133, Stro-1, nestin and the presence of SP within the WCP of HBM, suggests that expressed HBM may be a novel source of putative stem/progenitor cells. If proven, these valuable cells can be obtained non-invasively from lactating women, thereby creating a plentiful and ethically acceptable source of stem cells for use in a wide-range of mammary gland and stem cell biology applications.

Using the above stem/progenitor cell markers, we attempted to establish the presence of haemopoietic, mammary and mesenchymal stem cells through a range of well controlled culture conditions. However, we had been unable to culture any of these cells, which may reflect still suboptimal culture systems employed here, and may require in vivo models for further studies.

Satish et al, found a very high expression of 96.8% of CD29+ cells in the cellular component of HBM after they have been passaged in DMEM between two to six times while observing a comparatively lower expression of CD29 expressed by 10–15% of cells directly isolated from HBM [16], which is similar to our data. This indicated that either their culture conditions favoured the growth of the mesenchymal cells present or it coerced the CD29- cells to express β1integrin. We found markers of mesenchymal cells through the presence of Stro-1 in a quarter of cells in WCP of HBM and positive mRNA expression of osteonectin, alkaline phosphatase and osteopontin through RT-PCR. However, under carefully controlled conditions, we were unable to establish any adherent colonies despite either plating WCP or selected Stro-1 positive cells in standard MSC growth conditions at very low and high densities as compared to human fetal MSC.

The differences between Satish et al and our findings could be due to different culture conditions employed, such as the use of allogeneic human serum in contrast to our use of fetal bovine serum used routinely for culture of MSC in our laboratory [43], [44], [45]. Alternatively, the starting source of HBM may account for the differences observed here as Satish et al had isolated MSC from HBM obtained within five days of delivery while we utilised more mature milk between one to twelve months of delivery. Therefore, it is possible that the MSCs are shed into HBM in the earlier phase of lactation, but may not be present later on when lactation becomes established. Satish et al hypothesise that the source of these putative MSC may be the myoepithelial cells through the combined findings of CK5 [21] and smooth muscle actin [16] separately. Although epithelial-mesenchymal transition is usually characterized by the loss of cell adhesion proteins, myoepithelial cells, epithelial to mesenchymal transition-derived cells, genuine mesenchymal cells still share common markers [46], for instance CD29, making it difficult to ascertain if EMT has truly taken place. A definitive surface marker for epithelial cells would be helpful for dual-sorting alongside CD29 before culture might be useful in establishing EMT in the various culture systems involving the heterogenous cellular component of HBM.

We have previously demonstrated that the expression of nestin in WCP co-segregated with cells which can exclude Hoechst dye [21]. In this report, we affirmed the finding of SP in HBM in all 53 individuals. Comparisons made between the expressions of various markers present on SP and NSP suggested a difference between the two populations. The finding of primitive markers EPCAM and nestin and the lower expression of the mature epithelial marker CK18 in the SP fraction of WCP suggest the possibility of the presence of MaSC. While we were able to sort out cells that express stem cell markers; namely the side-population as well as CD133 cell surface marker, the failure to demonstrate self-renewal in current culture conditions including the well described mammosphere system suggest that they are unlikely to be mammary stem cells. However, this could still be due to the inefficiencies of the current culture system, and may require an *in vivo* model to further this line of investigations. While we are unable to conclusively state the absence of MaSC in HBM, it is worthwhile noting that if they are present, they do not conform to the established culture parameters and may require a switch-on/off mechanism.

In our study, we demonstrated the possible presence of stem/progenitor compartment of HBM. However, we were unable to culture them despite employing a range of routinely used established cell culture conditions, and may reflect sub-optimal culture conditions. In conjunction with the recent isolation of MSC in colostrums when cultured with human umbilical cord blood serum [16], we propose that continued research in this emerging area may lead to the identification of novel population of stem cells in HBM. Further work with appropriate in vivo model may shed more light in this area.

## Materials and Methods

### Samples collection and processing

Ethics Statement: All human tissue collection for research purposes was approved by the Domain Specific Review Board of National University Hospital. In all cases, patients gave separate written consent for the use of the collected tissue.

Forty-one breastfeeding women (duration of lactation – 1 to 12 months) were recruited to participate in the study. All samples were collected and processed within four hours of expression.

Umbilical cord blood was collected at normal deliveries at full term pregnancies and mono-nuclear cells recovered through density centrifugation, and used as controls for methylcellulose assays (n = 2).

Bone marrow derived human fetal MSC and mononuclear cells from peripheral blood were isolated as previously described [47], [48] were used as controls for RT-PCR.

### Isolation of cellular component of human breast milk

Cells were isolated from breast milk by centrifugation at 400 g for 10 min. The cellular component pelleted down was washed thrice with RPMI before use in culture and molecular work.

### Isolation of mono-nuclear cells from umbilical cord blood

Mononuclear cells from umbilical cord and peripheral blood were isolated as follows.

The umbilical cord blood was diluted 4-fold with 1X PBS (Gibco, Singapore), and layered over Ficoll 1077 (Invitrogen, Singapore) and centrifuged at 400 g for 35 min in a swinging-bucket rotor with the brakes off. The top layer of plasma was removed before collecting the mononuclear cells at the interphase, which were then washed twice with RPMI 1640 (Gibco).

### Reverse transcription polymerase chain reaction

Whole RNA was extracted from cells using the RNAeasy kit (Qiagen, Hilden, Germany) according to the manufacturer's instructions. The isolated DNAse-treated RNA was quantified with a spectrophotometer (Beckman, CA, USA) and 1 µg reverse-transcribed using Sensiscript RT kit (Qiagen) and oligo-dT18 primers (Proligo, Singapore) at 25°C for 15 min, 42°C for 60 min and 72°C for 15 min. Negative controls, without reverse transcriptase, were performed for each RNA sample to ensure absence of DNA contamination. For PCR amplification reactions, conditions used were 94°C for 2 min, 30–40 cycles of 94°C for 15 s, 60°C for 15 s, 72°C for 60 s and a final extension at 72°C for 4 min. The primers used for PCR amplification were as follows: D-glyceraldehyde-3-phosphate dehydrogenase: 5′AAGGACTCATGACCACAGTCCATG-3′ and 5′TTGATGGTACATGACAAGGTGCGG-3′; Nestin: 5′- GGTCAGTTCCTGAAGTTCACTCAG-3′ and 5′-CCTAGTACTATCGGGATTCAGCTG-3′; CK5: 5′-CGACAAGGTGCGGTTCCTG-3′ and 5′GCAGATTGGCGCACTG-3′; CK14: 5′- GATGACTTCCGCACCAAGTATGAG-3′ and 5′-TCAATCTCCAGGTTCTGCATGGTG-3′; CK18: 5′-AGAAATCTGAAGGCCAGCTTGGAG-3′ and 5′-TACCCTGCTTCTGCTGGCTTAATG-3′; CD34: 5′-GACACTGTGGACTTGGTCACCAG-3′ and GAGGAGGAAGCCATGGAGATCAG-3′; CD133: 5′-CCAAGTTCTACCTCATGTTTGG-3′ and 5′-ACCAACAGGGAGATTGCAAAGC-3′; Osteonectin: 5′-ATTTGATGATGGTGCAGAGGAA-3′ and 5′-GGTGGTTCTGGCAGGGATTT-3′; Alkaline Phophatase: 5′-CAGGCTGGAGATGGACAAGTTC-3′ and 5′-GGACCTGGGCATTGGTGTT-3′; Osteopontin: 5′-CCTGCCAGCAACCGAAGTT-3′ and 5′-CACTATCACCTCGGCCATCA-3′; Musashi-1: 5′-GTGTGAGGTGCTTAACCTATCAGC-3′ and 5′-ACAGATGTGGAGAGAAGAGACACC-3′; Neurofilament-M: 5′-CTTCAGCCAGTCCTCGTCCC-3′ and 5′-TCCTCCAGGTGGTCCGAGTC-3′.

### Flow cytometry

For the indirectly-labelled antibodies, cells were incubated with 5% bovine serum albumin (Sigma-Aldrich, Singapore), 20% goat's serum (Vector Laboratories, CA, USA) for 45 min at room temperature. Cell samples used for staining of intracellular antigens were fixed with 1 ml of methanol for 10 min at −20°C and then incubated in blocking solution as above with 0.5% Nonidet P40 (Roche, Basel, Switzerland) added before staining. Cells were suspended in 100 µl of the blocking solution and 5 µl of the primary antibody is added. The cells were incubated for 30 min at room temperature before washing twice with staining buffer (0.5% BSA/2 mM EDTA in 1X PBS) by centrifugation at 400 g for 5 min. Cells were resuspended and incubated for 15 min at room temperature in the dark in 488 AlexaFluor-labelled goat anti-mouse antibody:blocking solution (1∶200). For directly labelled antibodies, cells were suspended in 100 µl of staining buffer and 10 µl of fluorochrome-labelled antibody added into the suspension. After an incubation of 20 min in the dark, the cells were washed twice with staining buffer. The cells were resuspended in 1 ml staining buffer for analysis.

Analysis was carried out using CyanTM ADP analyzer (Dakocytomation) and results analysed with Summit 4.2.

### Fluorescent-activated cell sorting

Hoechst staining was performed as previously described [21]. Briefly, cells were washed from breast milk with warmed RPMI (Gibco). Cells were then resuspended in RPMI with Hoechst 33342 (Sigma) at a final concentration of 2.5 µg/ml and incubated for 1 hr at 37°C. In the control samples, verapamil (Sigma-Aldrich) was added (final concentration, 50 µg/ml) and after incubation, samples were washed and resuspended in HBSS (Gibco) supplemented with 5% FBS. Cells were stained with 1 µg/ml propidium iodide (Sigma-Aldrich) for viability studies.

Cells from breast milk were incubated with phycoerythin conjugated CD133/2 antibody (Miltenyi, Bergisch Gladbach, Germany), diluted 1∶10 in staining buffer (0.5% BSA/2 mM EDTA in 1X PBS). The cells are washed twice with staining buffer after incubation for 20 min at 37°C. Isotype controls were in place.

Cells from HBM were suspended in 100 µl of the blocking solution and 5 µl of Stro-1(Chemicon, MA, USA) is added. The cells were incubated for 30 min at room temperature before washing twice with staining buffer (0.5% BSA/2 mM EDTA in 1X PBS) by centrifugation at 400 g for 5 min. Cells were resuspended and incubated for 15 min at room temperature in the dark with AlexaFluor-488-labelled goat anti-mouse antibody:blocking solution (1∶200). Thereafter, the cells are washed once with staining buffer before being incubated in staining buffer supplemented with 5% FBS on ice until sorting is completed.

All samples were analyzed and sorted by flow cytometry using FACSAria and the software, FACS diva (Becton Dickinson, NJ, USA).

### Cell culture

#### Mammosphere culture

Cells from breast milk as well as a breast adenocarcinomoma cell line, MCF-7 were grown in a serum-free mammary epithelial growth medium (Biowhittaker, ME, USA), supplemented with B27 (Invitrogen), 20 ng/ml epidermal growth factor (EGF) (Sigma-Aldrich), 20 ng/ml basic fibroblast growth factor (bFGF) (BD Biosciences, NJ, USA) and 4 µg/ml heparin (Sigma-Aldrich). Cells were plated on ultralow attachment plates at various densities of 1, 100, 1,000 and 3,000 cells per well of a 96 well plate. Varying concentrations of EGF (20–100 ng/ml), bFGF (20–100 ng/ml), FBS (1–20%) and conditioned medium (1–80%) were used in the optimisation of mammosphere medium for culture.

#### Methylcellulose culture

Cells were placed in HSC-CFU lite with Epo (Miltenyi) at 1,000 to 3,000 cells per ml as per manufacturer's instructions. Briefly, individual specimens were mixed with 3 ml of methylcellulose containing bovine serum, stem cell factor, granulocyte-macrophage colony-stimulating factor, interleukins-3 and 6, erythropoietin, and plated in 35-mm tissue culture dishes in duplicates for 2 weeks.

#### Mesenchymal growth medium (D10)

Cells were cultured in Dulbecco's modified Eagle's medium (DMEM)-Glutamax (Gibco) supplemented with 10% FBS, 1X penicillin/streptomycin (Gibco).

#### Colony-forming unit-fibroblast assay

Sorted cells were cultured in D10 medium at a cell density of 4 cells per cm^2^ as per Zhang et al, [43] for two weeks, with medium change every three days. Colonies formed are then visualised by staining with 3% crystal violet in methanol for 10 minutes.

#### Bone differentiation medium

MSCs were plated at a cell density of 2×10^4^ cells per cm^2^ and cultured in D10 supplemented with 10 mM β-glycerophosphate, 10^−8^M dexamethasone and 0.2 mM ascorbic acid for two weeks, with medium changed every three to four days.

#### Von Kossa staining

Cells were washed gently with PBS before being fixed with 4% formalin for one hour. After washing twice with distilled water, the cells were stained with freshly made silver nitrate (Sigma-Aldrich) for ten minutes in the dark, exposed to sunlight for 30 minutes, before washing twice with distilled water.

#### Alizarin red staining

Cells were washed and fixed as above, and stained with 40 mM alizarin red (pH 4.2) for ten minutes at room temperature. Thereafter, they are washed thoroughly with PBS until clear.

### Statistical analysis

Parametric data are shown as mean± SEM, and analyzed using Student's t-Tests. A p <0.05 was considered significant.
